# Biomass residues as twenty-first century bioenergy feedstock—a comparison of eight integrated assessment models

**DOI:** 10.1007/s10584-019-02539-x

**Published:** 2019-09-10

**Authors:** Steef V. Hanssen, Vassilis Daioglou, Zoran J. N. Steinmann, Stefan Frank, Alexander Popp, Thierry Brunelle, Pekka Lauri, Tomoko Hasegawa, Mark A. J. Huijbregts, Detlef P. Van Vuuren

**Affiliations:** 1 Department of Environmental Science, Institute for Water and Wetland Research, Radboud University, P.O. Box 9010, 6500 GL Nijmegen, The Netherlands; 2 PBL Netherlands Environmental Assessment Agency, P.O. box 30314, 2500 GH The Hague, The Netherlands; 3 Copernicus Institute of Sustainable Development, Utrecht University, Heidelberglaan 2, 3584 CS Utrecht, The Netherlands; 4 IIASA, International Institute for Applied Systems Analysis, Schlossplatz 1, A-2361 Laxenburg, Austria; 5 PIK Potsdam Institute for Climate Impact Research, P.O. Box 60 12 03, 14412 Potsdam, Germany; 6 CIRAD, UMR CIRED, F-94736 Nogent-sur-Marne, France; 7 Center for Social & Environmental Systems Research, National Institute for Environmental Studies, 16-2 Onogawa, Tsukuba, Ibaraki, 305-8506 Japan

**Keywords:** Bioenergy, Biomass, Residues, Integrated assessment model, Supply, Availability

## Abstract

In the twenty-first century, modern bioenergy could become one of the largest sources of energy, partially replacing fossil fuels and contributing to climate change mitigation. Agricultural and forestry biomass residues form an inexpensive bioenergy feedstock with low greenhouse gas (GHG) emissions, if harvested sustainably. We analysed quantities of biomass residues supplied for energy and their sensitivities in harmonised bioenergy demand scenarios across eight integrated assessment models (IAMs) and compared them with literature-estimated residue availability. IAM results vary substantially, at both global and regional scales, but suggest that residues could meet 7–50% of bioenergy demand towards 2050, and 2–30% towards 2100, in a scenario with 300 EJ/year of exogenous bioenergy demand towards 2100. When considering mean literature-estimated availability, residues could provide around 55 EJ/year by 2050. Inter-model differences primarily arise from model structure, assumptions, and the representation of agriculture and forestry. Despite these differences, drivers of residues supplied and underlying cost dynamics are largely similar across models. Higher bioenergy demand or biomass prices increase the quantity of residues supplied for energy, though their effects level off as residues become depleted. GHG emission pricing and land protection can increase the costs of using land for lignocellulosic bioenergy crop cultivation, which increases residue use at the expense of lignocellulosic bioenergy crops. In most IAMs and scenarios, supplied residues in 2050 are within literature-estimated residue availability, but outliers and sustainability concerns warrant further exploration. We conclude that residues can cost-competitively play an important role in the twenty-first century bioenergy supply, though uncertainties remain concerning (regional) forestry and agricultural production and resulting residue supply potentials.

**Electronic supplementary material:**

The online version of this article (10.1007/s10584-019-02539-x) contains supplementary material, which is available to authorized users.

## Introduction

Model-based projections show that modern bioenergy^1^ could become one of the largest sources of energy over the course of the twenty-first century, replacing fossil fuels and hence contributing to climate change mitigation (Clarke et al. 2014; IRENA 2014; Rose et al. 2014; Smith et al. 2014; Creutzig et al. 2015; van Vuuren et al. 2016; Bauer et al. 2018; Rogelj et al. 2018). At present, modern bioenergy provides about 24 EJ per year or 4.2% of the global primary energy supply (IEA 2018). This share may increase to 10–35% (75–245 EJ/year) of the global primary energy supply by 2050 and to 10–50% (70–325 EJ/year; with low agreement on 300+ EJ/year) by 2100 (Chum et al. 2011; Rose et al. 2014; Smith et al. 2014; Creutzig et al. 2015; van Vuuren et al. 2016; Bauer et al. 2018).

Two generations of modern bioenergy are distinguished. The first generation of bioenergy is based on food crops. Second-generation bioenergy feedstocks include lignocellulosic bioenergy crops (i.e. cultivated fast-growing grasses or trees), residues, and wastes (Antizar-Ladislao and Turrion-Gomez 2008). Second-generation bioenergy is projected to supply a large share of future bioenergy use through advanced biofuels, electricity, and heat (Rogner et al. 2012; Rose et al. 2014; van Vuuren et al. 2016). Growing food or lignocellulosic crops for bioenergy can lead to competition for land with agriculture or natural areas, thus potentially threatening food security (Hasegawa et al. 2015) and biodiversity (Evans et al. 2015), and can increase net GHG emissions as a result of deforestation, foregone sequestration, or fertiliser use (Elshout et al. 2015; Creutzig et al. 2015; Albanito et al. 2016; Daioglou et al. 2017). Agricultural and forestry residues, on the other hand, are widely considered a promising and inexpensive bioenergy source (Carriquiry et al. 2011) with no or limited allocated land use and therefore generally low climate change, biodiversity, and other environmental impacts (Smith et al. 2014; Creutzig et al. 2015), if residue removal rates are low enough to sustain carbon stocks, soil fertility, and other ecological functions (Raffa et al. 2015; Repo et al. 2015). Hence, residue use as a bioenergy feedstock is commonly encouraged (e.g. EU Directives 2009/28/EC and 2015/1513).

Agricultural residues include harvest and processing residues, while forestry residues include, i.a., logging, thinning, and processing residues (for an overview, see Smith et al. 2014; Creutzig et al. 2015). Using a range of methodologies (explored in detail in section 3.4), the mean estimated residue availability^2^ in 2050 for primary energy is 36 EJ/year for agricultural residues (10–55 EJ/year minimum-maximum range; excl. animal dung), 25 EJ/year (5–50 EJ/year) for forestry residues, and 61 EJ/year (12–76 EJ/year) combined (Fischer and Schrattenholzer 2001; Hoogwijk et al. 2003; Smeets and Faaij 2007; Smeets et al. 2007; Hakala et al. 2009; Gregg and Smith 2010; Haberl et al. 2010, 2011; Cornelissen et al. 2012; Rogner et al. 2012; Lauri et al. 2014; Searle and Malins 2015; Daioglou et al. 2015). The future economic and ecological *availability* of residues as primary energy source and its drivers and sensitivities have thus been extensively studied in previous work. However, it has not been explored what amount of residues can cost-competitively be *supplied* as primary bioenergy feedstock when competing against other bioenergy feedstocks to meet a given bioenergy demand or what drivers determine the quantity of residues supplied for bioenergy and via what mechanisms. All of which are essential in understanding what role residues could have as an energy source, including in climate change mitigation pathways (Clarke et al. 2014; Rogelj et al. 2018).

In this study, we explore the quantity of biomass residues supplied (i.e. dispatched) for energy use and their share in the total bioenergy supply over the course of the twenty-first century, in eight integrated assessment models (IAMs; see section 2.2). We compare model structure, assumptions, and outcomes across the IAMs. Residues here constitute agricultural and forestry residues. Supply potential is subject to ecological and economic constraints. We use diagnostic scenarios with exogenous bioenergy demand or prices to analyse how the supplied quantity of residues and share of residues within total bioenergy supplied depend on four drivers that could be directly or indirectly influenced by climate and energy policy: (1) the demand for modern bioenergy, (2) pricing GHG emissions, (3) land protection efforts, and (4) the price of biomass. We also compare the quantity of residues supplied in IAMs with estimates of residue availability in literature, to evaluate if the role of residues as twenty-first century energy source in different IAM scenarios matches expected availability.

## Methods

### Model selection

The IAM projections used in this study were developed within the context of the Energy Modelling Forum 33 Bioenergy Study (EMF-33). The EMF-33 study aims to understand, analyse, and improve modelling of biomass supply and demand within IAMs (Rose et al., *this issue*). We analysed projections of all eight IAMs within EMF-33 that reported quantities of biomass residues supplied for primary energy, viz., the AIM, BET, DNE21+, GCAM, GLOBIOM, GRAPE, IMAGE, and NLU models (see Table S1). The NLU and GLOBIOM models are not IAMs *sensu stricto*, but rather economic land-use models that focus on agriculture and forestry, respectively.

### Model description

IAMs are designed to explore different future energy and land-use consumption and production patterns and their associated environmental impacts. For bioenergy, they describe both demand and supply. This study focuses on the supply side. We use scenarios in which either the demand for modern bioenergy or the price of biomass is exogenously set and harmonised across all the IAMs (section 2.3) and determine the cost-optimal quantity of residues supplied. To meet an exogenous bioenergy demand, bioenergy feedstocks—including residues—compete with each other based on costs. At an exogenous biomass price, feedstock is supplied for bioenergy if the feedstock’s costs are lower than the exogenous biomass price.

Key characteristics of the individual IAMs in terms of their representation and modelling of residue supply and residue costs are given in Table 1. All models include both agricultural and forestry residues, except GLOBIOM, which only models forestry residues for energy use. IMAGE and GRAPE also include municipal solid waste as residues. Besides residues, second-generation bioenergy feedstocks include lignocellulosic bioenergy crops in all models, as well as managed forests and plantations in the BET, GLOBIOM, and NLU models. Residue supply potential is determined endogenously in most IAMs, based on agricultural and forestry production, while it is exogenously set for DNE21+ and GRAPE. Bioenergy demand does not stimulate residue production as residues are considered by-products and not co-products in all studied IAMs, except GLOBIOM in which demand for residues can (co-)incentivise additional roundwood harvesting.

**Table 1:** Key characteristics of the integrated assessment models (IAMs) included in this study

	Residue types	Residue supply potential	Residue supply constraints^a^	Residue supply curve	Residue cost components	Conversion restrictions^b^
AIM	ARFR	Endogenous via *input/output structure*	Ecological, economic *combined effect: 50% of total residues available*	Endogenous via *input/output structure*	Processing	Electricity, biofuels
BET	ARFR MSW O	Endogenous via *GLUE model*^*c*^ *component*	Ecological, economic *accounted for* via *exogenous supply curve*^*d*^	Exogenous^d^	Collection	Electricity, biofuels, biogas
DNE21+	AR FR	Exogenous^c^	Ecological, economic *accounted for* via *economic potential in GLUE*^*c*^	Exogenous^c^	Collection, transport, processing	Electricity, biofuels, hydrogen,solids
GCAM	AR FR	Endogenous *based on agricultural and forestry production*	Ecological *unspecified fraction remains on land*	Endogenous	Collection, transport, processing	None
GLOBIOM	FR^e^	Endogenous *based on forestry production*	Ecological: *min. 50% of residues is left on the field* economic: *competition with fibre*	Endogenous	Collection, transport^f^	None
GRAPE	AR FR	Exogenous^g^	Ecological, economic *accounted for* via *exogenous supply potential*^g,h^	Exogenous^g^	Collection, transport, processing	ELECTRICITY, heat
IMAGE	AR FR MSW	Endogenous^i^ *based on agricultural production and timber demand*	Ecological: *30% of residues is left on the field*^j^ economic: *feed and traditional bioenergy first*	Endogenous^i^	Collection, transport,processing	Electricity, heat, hydrogen
NLU	AR FR	AR: endogenous *based on agricultural production* FR: exogenous^k^	Ecological, economic *combined effect: max. 30% of AR and 50% of FR available for energy*^l^	None *available residues used for bioenergy*^l^	Transport	None

*AR*, agricultural residues (incl. processing/secondary residues; excl. animal dung); *FR*, forestry residues (incl. processing/secondary residues); *MSW*, municipal solid waste; *O*, other (kitchen refuse, sewage sludge); Notes: ^a^Ecological constraints (i.e. leave residues on land to maintain soil fertility, stability, and/or carbon stocks) and economic constraints (i.e. alternative non-energy uses of residues); ^b^Restrictions that limit energy use of residues to certain sectors; ^c^Based on Yamamoto et al. (2001); ^d^Based on exogenous supply costs derived from Daioglou et al. (2015); ^e^GLOBIOM includes beyond harvesting and processing residues: recycled wood, stump removal, and additional roundwood extraction for bioenergy. ^f^Harvest costs 5-40US$/m^3^ based on G4M, transport costs via price elasticity function; ^g^Based on exogenous supply costs derived from Rogner et al. (2012); ^h^Residues supplied for modern bioenergy further constrained by competition with residue use for traditional bioenergy; ^i^Residue supply potential and supply curves are endogenous for forestry and agricultural residues in IMAGE, but are exogenous for MSW, see c; ^j^Mass constraint per hectare, which globally aggregates to 30% of residues left on the field; ^k^Smeets et al. (2007); ^l^In NLU, agricultural residues are first used to meet feed demand; of the remaining residues, 40% stays on the field, 30% goes to pulp and construction materials, and 30% is (always) used as bioenergy feedstock. In NLU, 50% of forestry residues are used for pulp and construction material and 50% are (always) used as bioenergy feedstock. Residue costs are determined endogenously

In all models, supply potential is constrained by ecological constraints (i.e. requirements to leave residues on agricultural land or forestland to maintain soil fertility and/or carbon stocks, and/or to prevent erosion), as well as economic constraints (i.e. alternative residue uses, for non-energy purposes; for details per model see Table 1). Residue costs are based on collection, transport, and/or processing and are related to the supply of different bioenergy feedstocks, through supply curves^3^ (except in NLU, see section 3.2). Most models have endogenous residue supply curves that are determined as part of the model run. However, BET, DNE21+, and GRAPE have fixed, exogenous supply curves derived from literature. All models assume residues cause no GHG emissions other than supply chain emissions; because biogenic carbon emissions are considered GHG neutral, residue extraction is ecologically constrained, and no direct land-use change emissions are allocated to residues.

### Scenario selection and model comparison

The studied IAMs were all run according to EMF-33 scenarios; for an overview of all EMF-33 biomass supply scenarios, specification details, and rationale, see Rose et al. (this issue). The scenario subset used here includes the following: (1) scenarios with an exogenous demand for second-generation primary bioenergy that increases linearly from the modelled demand in 2010 to 100, 200, 300, or 400 EJ/year by 2100 (scenario B100/200/300/400; see Table S2), either with or without GHG pricing and either with or without land protection; and (2) scenarios with an exogenous fixed biomass price of 3, 5, 9, or 15 US$_2005_ /GJ at farm gate/roadside (scenario PB3/5/9/19; see Table S2), either with or without GHG pricing. GHG pricing means that emitting GHGs has a price of 20 US$_2005_/t CO_2_eq. in 2020 with a 3% annual increase. This GHG price is applied to all major GHGs (CO_2_, CH_4_, and N_2_O) including all land-use-related GHG emissions. Land protection means that on top of default model constraints on land availability,^4^ further areas are to remain in or transform to a natural state and are not available for human land uses such as agriculture. All scenarios are based on reference socioeconomic assumptions; i.e. socioeconomic and technological parameterisation of the scenarios is based on SSP2 (Popp et al. 2017) and is run from 2005 to 2100.

The purpose of these diagnostic scenarios is not to make definite projections of future residue use. Rather, these scenarios allowed us to compare the supplied quantity of residues across models when “forced” to supply bioenergy under exogenous bioenergy demand or biomass prices. These scenarios help to determine what role residues could play in meeting total bioenergy demand, alongside purpose-grown feedstocks like lignocellulosic bioenergy crops, and to show what dynamics underlie the quantity of residues supplied in the different models. The scenarios also allow assessing the sensitivity of the quantity of residues supplied to four main drivers: (1) bioenergy demand, (2) GHG emission pricing, (3) land protection, and (4) biomass prices. We analysed absolute quantity of residues supplied and the share that residues form in the total amount of second-generation bioenergy supplied.

Additionally, we used variance decomposition analysis to provide an indication of how individual drivers contribute to the modelled quantity of residues supplied. Taking the quantity of residues supplied across scenarios as dependent variable, we performed an ANOVA to derive the sum of squares (SSQ) for the factors Bioenergy Demand and GHG Pricing, and for the residuals, which represent model variability between the included IAMs. The SSQs of both factors and the residuals were divided by the total SSQ, yielding the variance attributable to these factors and residuals. Log-transformed supplied residue values, or logit-transformed shares of residues in the total bioenergy supply, were used to minimise the influence of outliers.

### Literature analysis of future residue availability

We compared the *quantity of residues supplied* in IAMs to the *expected availability of residues* estimated in literature, to determine if the role of residues in IAM scenarios fits within the expected availability of residues. We define residue availability here as the technical potential (IPCC terminology; Chum et al. 2011) or equivalently the available potential (Daioglou et al. 2015) of residues, which accounts for ecological constraints (i.e. preserving soil quality, carbon storage, and sometimes biodiversity) and economic constraints (i.e. alternative uses of residues) on residue supply. Our literature analysis includes all peer-reviewed studies published since 2000 that estimate the global available/technical potential of forestry and/or agricultural residues over the course of the twenty-first century, and specifically in 2050. We consider the default available/technical potential reported in these studies. If no default is defined, we use the mean of reported values. We look at the minimum-maximum range per study, based on the lowest and highest reported estimates of residue availability in 2050 across sensitivity tests and scenarios.

We distinguish two types of studies. First, studies with a bottom-up approach that directly estimate residue availability from expected trends in population size, diet and consumption patterns, and ultimately agricultural and/or forestry production. And second, studies with top-down macro-economic drivers that estimate residue availability based on macro-economic, IAM, or IAM-component model results. While some of these latter estimates are based on the same or similar models that were used in this study, it is relevant to compare our diagnostic scenario-based results against their *projected* residue supply. This comparison also serves as a further plausibility check.

## Results

### The importance of residues as bioenergy feedstock

**Fig. 1:**
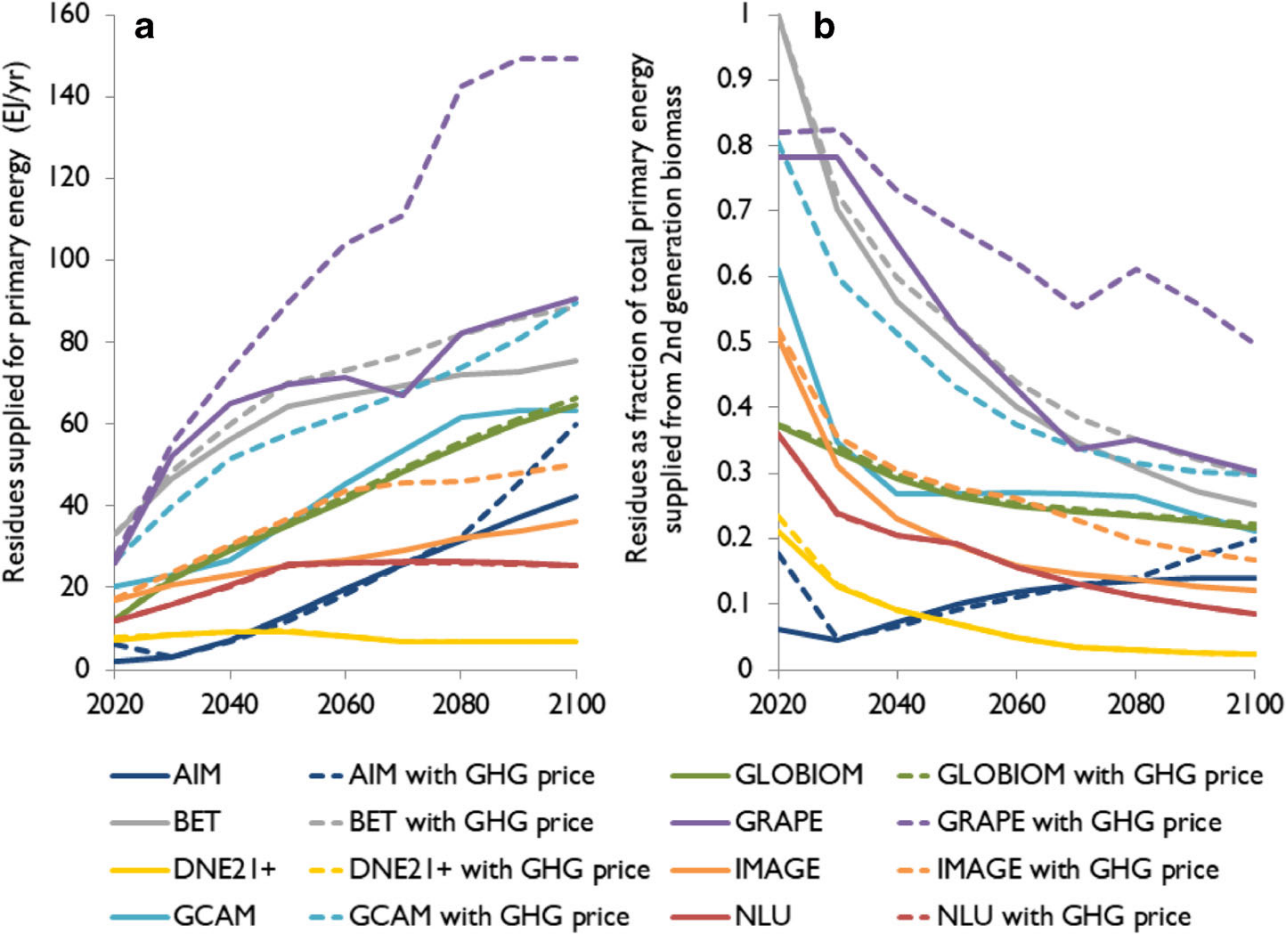
Quantity of residue supplied for primary energy (EJ/year) at an exogenous demand for second-generation bioenergy that increases linearly from 2010 levels to 300 EJ/year by 2100, with and without GHG pricing (**a**) and residues as share of total second-generation biomass use for primary energy under the same scenarios (**b**). Dotted lines may underlie their respective solid line

Figure 1a shows the quantity of residues supplied in two scenarios: (1) a scenario with an exogenous demand for second-generation primary bioenergy that increases linearly from 2010 levels to 300 EJ/year by 2100 and (2) the same scenario including a price on emissions (scenarios B300 and B300C in Table S2, respectively). Figure 1b presents the share of residues as part of total supplied second-generation bioenergy for these same scenarios. Analogous figures with exogenous bioenergy demands of 100, 200, and 400 EJ/year can be found in the supplementary materials (Figure S1-S3). Both the quantity of residues supplied and the share of second-generation bioenergy covered by residues vary widely across the studied IAMs at a given exogenous bioenergy demand level. In the 300 EJ/year demand scenario for example, the quantity of residues supplied in 2100 ranges from 7 to 91 EJ without GHG pricing, and up to 151 EJ with GHG pricing. GHG pricing effects are limited in most models, as detailed in section 3.2.

Inter-model consensus is highest among IAMs with endogenous supply curves (i.e. AIM, GCAM, GLOBIOM, IMAGE) and the NLU model with 25–90 EJ supplied by 2100, covering 10–30% of bioenergy demand (Fig. 1). Meanwhile, the BET and GRAPE models have exogenously derived supply curves based on low exogenous residue costs and show the largest amounts of residues supplied for energy. DNE21+, on the other hand, has an exogenous supply curve based on higher costs and projects the lowest quantity supplied. DNE21+ and GRAPE also have exogenous residue supply potentials, which may further add to the more extreme outcomes of these models.

Beside model structure, two other sets of factors add to the observed variation in model outcomes. First, in models with endogenous residue supply potential (all models, except DNE21+ and GRAPE, see Table 1), agricultural and forestry production affect residue supply potential. We find that agricultural production varies around 20% among models^5^ (Figure S4), caused by 50% inter-model variation in livestock produced, 15% variation in food demand, 25% variation in food crop yields (Figure S5), and variation in the type of food crops produced. Forestry production and associated residue production even vary by a factor of ten among the models, with IMAGE on the low end and GLOBIOM and BET on the higher end (Figure S4). However, while crop yields, diet, agricultural production, and forestry production vary across models, and while these variables affect the residue supply potential in IAMs with endogenous supply potential, they are not consistently related to the quantity of residues supplied in these IAMs.

Second, definitions of residues, constraints, and costs vary between models (Table 1). GLOBIOM excludes agricultural residues, but reports outcomes that are in the middle of the inter-model range. GRAPE and IMAGE include municipal solid waste (MSW) as residues, which add about 10% to the supply potential in GRAPE, but a smaller amount in IMAGE. Ecological and economic constraints are present in all models, but vary, for instance, concerning the percentage of residues that should remain on the field, or the competing alternative uses of residues. The IAMs also vary with regard to the types of residue costs they include, i.e. collection, processing, and/or transport costs, and what economic sectors use residues for energy. While adding variation, these effects do not show a consistent effect across models on the quantity of residues supplied.

Despite variability between IAMs and across different exogenous demand levels, IAM outcomes show that residues generally form an important bioenergy feedstock, meeting 7–50% of bioenergy demand towards 2050, and 2–30% towards 2100, in the 300 EJ/year in 2100 scenario (B300). The absolute quantity of residues supplied grows over time, mostly driven by increasing exogenous bioenergy demand over time and eventually levels off. The share of residues in the total amount of second-generation bioenergy supplied nevertheless decreases over time as residue supply cannot keep up with increasing bioenergy demand. Remaining demand is met by lignocellulosic bioenergy crops and managed forest (Rose et al. this issue).

### Model drivers of residue supply

**Fig. 2:**
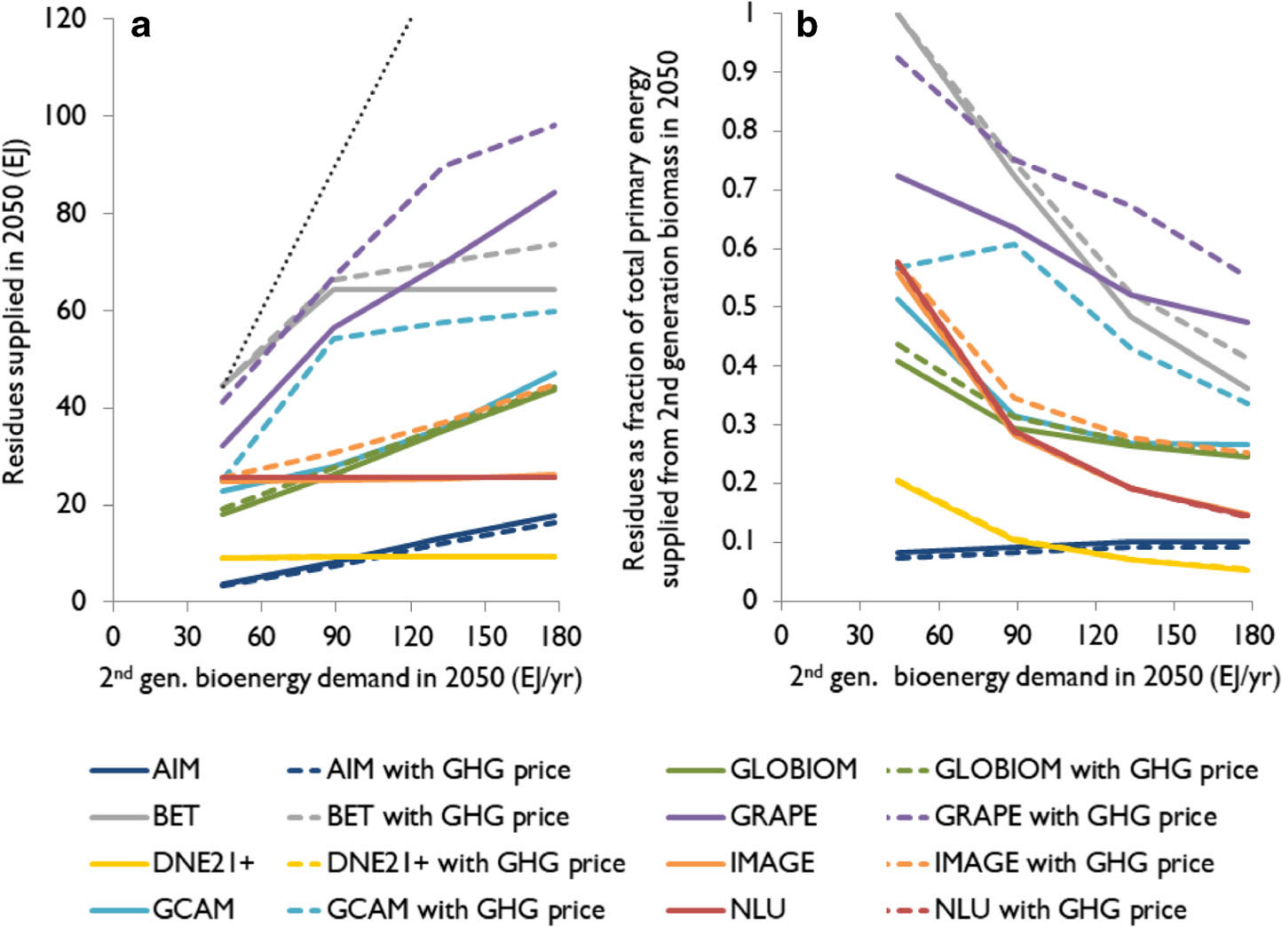
Quantity of residues supplied in the studied IAMs for the year 2050, across four scenarios with increasing exogenous bioenergy demand (to 100, 200, 300, and 400 EJ/year by 2100), with and without GHG pricing (**a**) and residues as share of total second-generation biomass use for primary energy across the same scenarios in 2050 (**b**). The black dotted line indicates residues meeting 100% of exogenous bioenergy demand

Figure 2 shows the global quantity of residues supplied in IAM projections in the year 2050, across four scenarios with increasing exogenous second-generation *bioenergy demand*. A higher exogenous bioenergy demand leads to a larger quantity of residues supplied (i.e. dispatched for energy) in most models^6^ (Fig. 2a). Bioenergy demand does not, however, directly stimulate the production of residues. Rather, a higher bioenergy demand increases bioenergy prices, which leads to more residues being taken off the field and/or more residues being diverted from other sectors towards bioenergy, leading to increased quantities of residues supplied for energy.

Nevertheless, the share of residues in second-generation bioenergy *decreases* with bioenergy demand (Fig. 2b). While residues are a relatively cheap feedstock and thus form a large share of total bioenergy at low demand, residue supply is more constrained than that of other feedstocks such as lignocellulosic bioenergy crops and does not keep up with demand. Constraints include the total volume of residues, which does not increase with bioenergy demand, as well as the amount of residues that can be diverted from the field (ecological constraints) and from other sectors (economic constraints; see Table 1). These patterns observed for 2050 are the same in other years (Figure S6).

In BET, GCAM, GRAPE, and IMAGE, *pricing GHG emissions* (20 US$_2005_/tCO_2_-eq. in 2020 plus a 3% annual increase) increases both the quantity of residues supplied and the share of residues in the bioenergy mix for the exogenous demand scenarios (Figs. 1 and 2, S1–3). The reason being that in the IAMs, residues lead to no or low (allocated) GHG emissions, compared with other bioenergy feedstocks like lignocellulosic bioenergy crops or wood from managed forests, which can require land-use change and lead to larger supply chain/lifecycle emissions. Therefore, GHG pricing does not increase residue costs much, but it does increase the costs of other bioenergy feedstocks. This makes residues a more favourable feedstock and incentivises taking residues off the field or diverting them from other sectors, thus expanding the energy use of residues. These dynamics are, however, limited or absent in AIM, DNE21+, GLOBIOM, and NLU.

Our *variance decomposition analysis* shows that the majority of variation in the quantity of residues supplied across IAMs and scenarios is attributable to differences in IAMs (82–93% variance explained; Table S4). Sources of this variation across IAMs are presented in section 3.1 and discussed in section 4.2. Substantially less variation is, however, attributable to the scenario components of exogenous bioenergy demand (5–16%) and GHG pricing (0–3%; Table S4). The ranges shown include analyses throughout the twenty-first century, for both the absolute quantity supplied and the share of residues in total bioenergy supplied (see Table S4 for examples for the years 2050 and 2100). When DNE21+ and NLU, which do not respond to exogenous bioenergy demand or GHG pricing, are excluded from this analysis, a larger part of the variation in residues supplied is explained by bioenergy demand (12–25%) and GHG pricing (0–12%), though inter-model differences still account for the majority of variation (63–88%).

*Land protection*, which is only modelled in IMAGE and GCAM, excludes economic activity from certain areas, making remaining land more expensive. This disproportionally increases the costs of land-intensive lignocellulosic bioenergy crops and increases overall bioenergy prices. Meanwhile, residue costs are *less* affected and residues thus become the more cost-optimal feedstock. The supplied quantity of residues to meet a given bioenergy demand therefore increases (Figure S7), as more residues are taken off the field or diverted from other sectors, incentivised by the increased bioenergy prices. This increase in energy use of residues is co-facilitated by higher yields and residue production on the scarcer and therefore more intensively managed agricultural/forestland. Ultimately, the effect of land protection on residue supply is similar and complementary to that of GHG emission pricing, until available residue supply levels off (Figure S7).

The effect of the *price of biomass* on the quantity of residues supplied is simulated in the GLOBIOM and IMAGE models using exogenous biomass price scenarios. In these models, the quantity of residues supplied is a consequence of complex relationships that, besides the biomass price, include residue costs, competition with other feedstocks, and food and timber market dynamics. Higher prices of second-generation biomass lead to larger quantities of residues supplied for energy (Figure S8), as there is incentive to take more residues off the field or divert them from non-energy sectors. This happens independently from the supplied quantity of lignocellulosic bioenergy crops, which also increases (Rose et al., this issue). The increase in residues supplied for bioenergy levels off at higher prices, as the maximum residue supply is reached under ecological and economic constraints (Table 1). These dynamics are hardly influenced by GHG pricing and land protection (Figure S8).

### Regional differences in the quantity of residues supplied

**Fig. 3:**
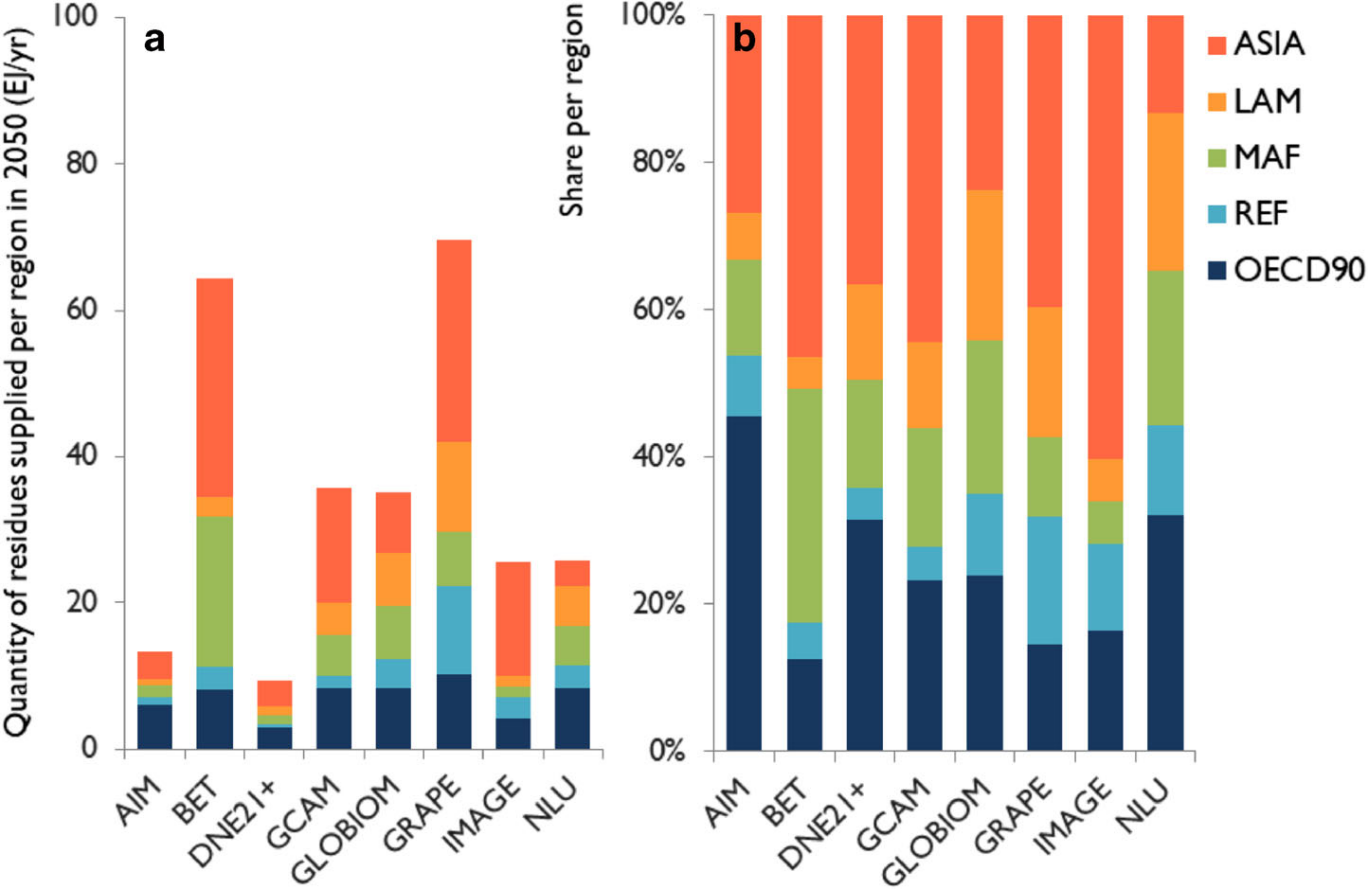
Quantity of biomass residues supplied for energy per region in 2050 in the scenario with an exogenous primary bioenergy demand of 300 EJ/year by 2100 (**a**) and the share of residues supplied for energy per region in 2050 in the same scenario (**b**). LAM, Latin America; MAF, Middle East and Africa; REF, reforming economies (former Soviet Union and Eastern Europe); OECD90, OECD member countries in 1990. For regional definitions, see Table S2

Figure 3 shows the absolute (Fig. 3a) and relative (Fig. 3b) quantities of residues supplied per region across the studied IAMs for 2050 in the 300 EJ/year exogenous bioenergy demand scenario. In most models, Asia supplies most residues for energy (24–60%), followed by the OECD90 countries, which form the largest supplier in NLU and AIM, and Africa (Fig. 3b; for region definitions, see Table S3). There are, however, large inter-model differences, for instance, the exact share of Asian supply, or the large role of African supply in BET and South American supply in GLOBIOM, GRAPE, and NLU. These differences are even larger in absolute terms (Fig. 3a), with, for example, Asian-supplied residues in BET and GRAPE equalling 80–320% of *global* supplied quantities in the other models. These patterns per model stay approximately the same when including GHG pricing, or considering different years or levels of exogenous bioenergy demand (Figure S9–11).

The large disagreement among IAMs on quantities of residues supplied per region can be explained by the following: (1) differences in model structure and assumptions, as explained in section 3.1 (see also Table 1), and (2) large inter-model differences in regional agricultural and forestry production (Figure S12 and Fig. 3) residue definition, specifically: GLOBIOM only includes forestry residues.

### Residues supplied in IAMs versus residue availability in literature

**Fig. 4:**
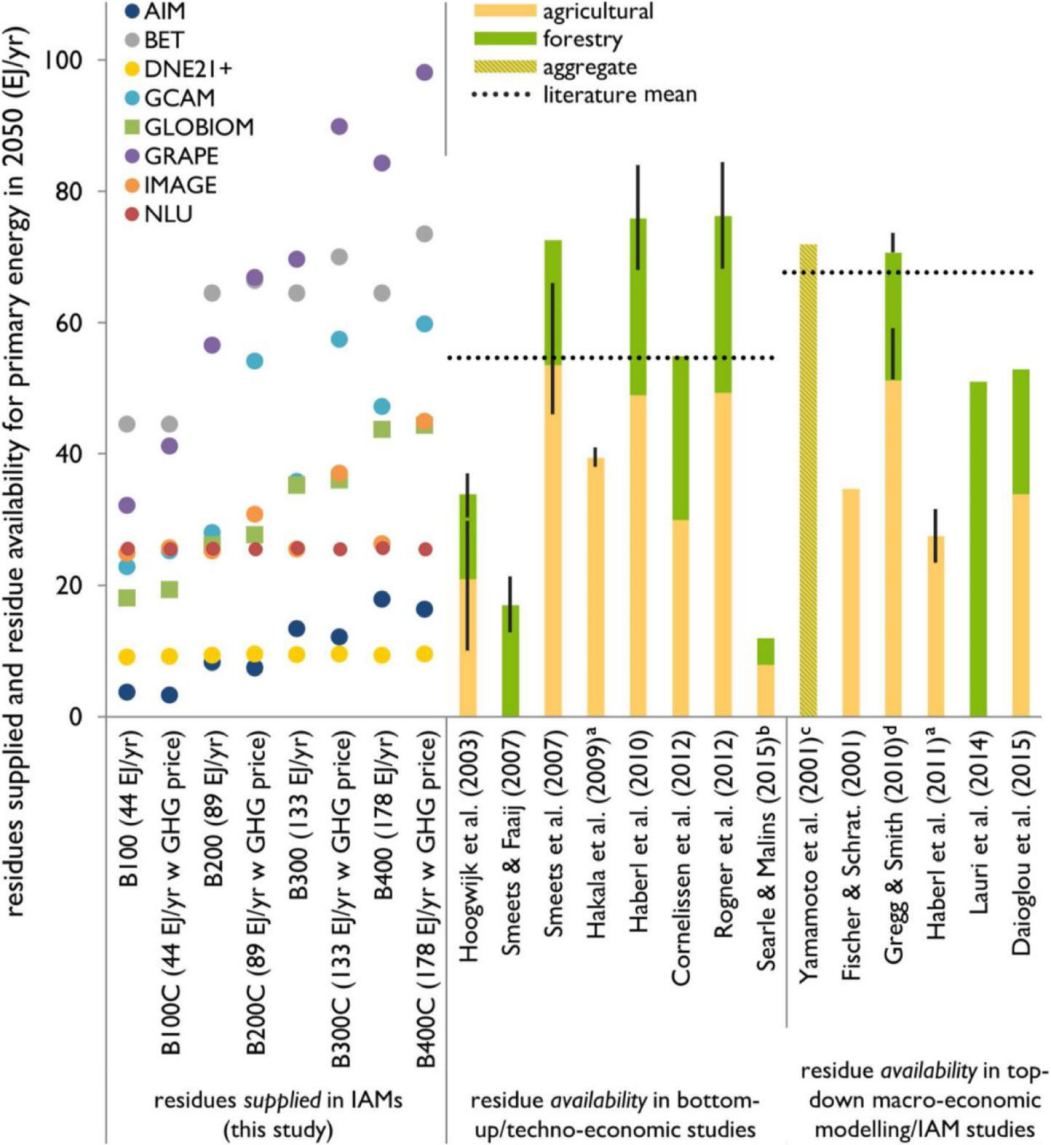
Comparison of the quantity of residues *supplied* for energy in 2050 as projected by the studied IAMs across scenarios against expected residue *availability* in 2050 in literature. GLOBIOM projections (in green *squares*) only include forestry residues. Literature means are calculated as the mean availability of agricultural residues plus the mean availability of forestry residues (both including processing/secondary residues). Error bars indicate minimum and maximum values where provided in literature. Notes: *a*, excludes processing residues; *b*, very strict sustainability criteria; *c*, includes animal dung; *d*, only subject to ecological constraints (i.e., no economic constraints)

Figure 4 shows a comparison of the modelled quantity of residues *supplied* in 2050 in all studied IAMs and exogenous bioenergy demand scenarios, against the expected residue *availability* in 2050, as estimated in literature. This comparison serves to determine if the role of residues in IAM scenarios fits within expected residue availability. Comparison against bottom-up estimates of availability is especially useful here, since these estimates are based on a different approach, independent of top-down or IAM modelling effects. Comparison against top-down macro-economic/IAM-modelled residue availability serves as a further plausibility check. While both IAMs and the literature estimates include ecological and economic constraints on residue supply potential, it is important to note that IAMs determine the cost-optimal, “competitive” quantity of residues supplied, while literature estimates consider total residue availability, under the constraints set. Supplied quantities can therefore certainly be lower than availability, but higher projections indicate that such residue use is infeasible.

Estimates of residue availability in bottom-up studies range 12–76 EJ/year in 2050, with a mean of 55 EJ/year, which is determined as the sum of mean agricultural and mean forestry residue availability. The wide range in residue availability can be explained by different methodologies, as well as differences in the definition of economic and ecological constraints. Early work by Hoogwijk et al. (2003) indicated a residue availability of around 34 EJ/year. Smeets and Faaij (2007), Smeets et al. (2007), Hakala et al. (2009), and Haberl et al. (2010), whose results were also part of the literature assessment by Rogner et al. (2012), reported larger availability of both agricultural and forestry residues. These studies look at the maximum realistically possible availability of residues for energy. In contrast, Searle and Malins (2015), and to a substantially lesser extent Cornelissen et al. (2012), include stricter sustainability constraints (i.e. no residue extraction from natural forests, 70% of agricultural residues unavailable) and estimate residue availability in 2050 to be 12 EJ/year—80% below this study’s literature average.

Studies based on top-down macro-economic modelling and IAMs report high residue availability estimates, with a mean of 68 EJ/year in 2050. However, there is large variation in this group of studies as well. Gregg and Smith (2010) report the largest residue availability, which can be explained by the fact that they estimate ecological potential only and do not include economic constraints (i.e. alternative uses of residues). Yamamoto et al. (2001) also report relatively large residue availability, but include animal dung, which is excluded in other studies. Excluding these two studies lowers the mean of residue availability estimates in top-down studies to 56 EJ/year in 2050, similar to the 55 EJ/year mean across bottom-up studies.

The quantity of residues supplied in the studied IAMs varies between and within scenarios, due to not only model assumptions and structure but also scenario-specific differences in bioenergy demand, biomass price, and GHG pricing. In most cases, the quantity of residues supplied in 2050 in IAMs is lower than that in the literature-estimated availability. This means that the relatively large role that the IAMs attribute to residues in meeting the potentially large future bioenergy demand generally seems possible based on our current understanding of future residue availability (including ecological and economic constraints).

It is, however, important to note that in scenarios with high exogenous bioenergy demand, and especially with GHG pricing, the quantity of residues supplied in the GRAPE, BET, and GCAM models can be higher than that of the literature-estimated residue availability. This can (in part) be explained by the high exogenous residue supply potential of the GRAPE and BET models, which is not in line with recent estimates of residue availability (Fig. 4), and by the relatively large agricultural production in the GCAM model. Furthermore, GLOBIOM only reports forestry residues and at high bioenergy demand only stays within the availability^7^ estimated by Lauri et al. (2014). The amount of residues supplied for energy in these models in these particular scenarios warrants further exploration, as it does not always match bottom-up or top-down estimates of residue availability.

## Discussion

### Model interpretation

We found that the quantity of residues supplied for bioenergy in the studied IAMs varies substantially, but meets anywhere from several percent to up to half of total second-generation bioenergy demand by 2050, and up to around 30% by 2100. As future bioenergy use is expected to be significant (Chum et al. 2011; Bruckner et al. 2014; Clarke et al. 2014; IRENA 2014; Rose et al. 2014; Smith et al. 2014; Creutzig et al. 2015; van Vuuren et al. 2016), biomass residues may play a large role in the twenty-first century energy supply. In terms of drivers of residue use, we found that a higher bioenergy demand or biomass price increases the quantity of residues supplied, though their effects level off at higher demand or prices. GHG pricing and land protection increase the costs of land, which in most models leads to increased residue use, at the expense of lignocellulosic bioenergy crops. These patterns and drivers of residue supply were similar across models and are well-understood, as IAMs allowed for explicit analysis of the cost dynamics that underlie them. Specific IAM results, however, differed markedly—with model differences explaining 82–93% of the variation in results, as discussed in section 4.2. Lastly, we found that in most IAMs and scenarios, supplied residue quantities in 2050 were found to be within literature estimates of residue availability. The large role IAMs attribute to residues in meeting bioenergy demand thus seems plausible. The feasibility of such large-scale residue use is discussed in section 4.3.

### Variability in IAM results

As summarised by our variance decomposition analysis, IAM outcomes varied significantly within scenarios at the global *and* regional scale, despite a shared storyline and scenario assumptions. Several factors contribute to the observed differences. First, models with exogenous residue supply potential and supply curves showed more extreme outcomes in the quantity of residues that is supplied for bioenergy. Models with endogenous supply potential and curves better captured residue supply dynamics and lead to more similar results in this study.

Second, structural inter-model differences in agricultural and forestry production and the assumed allocation of production over different end-uses affected the supplied quantity of residues and along with residue costs (see below) affected the calibration of supply curves. Crop yields, diets, agricultural production, and forestry production were, however, not correlated with the quantity of residues supplied across models. Previous work (Daioglou et al. 2015) showed that these variables may have counteracting effects. Modelling may be improved here by adding scenarios with harmonised assumptions on food and forestry product (i.e. timber and fibre) demand to deduce their influence. The dynamic relationship between agricultural/forestry production and residue supply, which is often assumed to be linear, could also be modelled in more detail, e.g. through crop-specific relationships. Furthermore, the role of residues in the total amount of bioenergy supplied can be investigated in sensitivity scenarios with different crop yields and diets, which are of key importance to the amount of land available for bioenergy crops (Gerssen-Gondelach et al. 2015; Stehfest et al. 2009).

Third, residue definitions and the assumed level and nature of ecological and economic constraints varied among models. As did residue costs, since residues form a diverse feedstock with a wide range of costs that depends strongly on local circumstances. These differences in constraints and costs contribute to inter-model variation, but do not show a consistent effect on residue supply dynamics. More detailed and harmonised constraints on residue supply^8^ and detailed cost components would advance our understanding of residue supply dynamics and the range of IAM outcomes.

### The feasibility of large-scale residue use

Quantities of residues supplied in IAMs that lie within estimated availability may be possible, but more general aspects of their feasibility still need addressing. First, IAMs assume that all residues—subject to ecological and economic constraints—are usable and substitutable, regardless of the exact type or origin of the residue. Quality and logistical constraints may, however, reduce the quantity of residues that can be used in reality or increase transport and processing costs. If transport distances to processing locations are for instance too large, high transport costs may render residue use infeasible, while transport emissions could make residue use undesirable (Portugal-Pereira et al. 2015).

Second, biomass residues have low or no additional land requirements and associated GHG emissions or competition with food (Smith et al. 2014; Creutzig et al. 2015). Nevertheless, for residues to be a truly sustainable feedstock, it is critical that (enhanced) residue extraction does not lead to erosion or losses in soil fertility, biodiversity, or carbon stocks (Lal 2005; Janowiak and Webster 2010; Lemke et al. 2010; Bouget et al. 2012; Lamers et al. 2013; Liska et al. 2014; Raffa et al. 2015; Poeplau et al. 2015; Repo et al. 2015). IAMs, as well as most studies on residue availability, include this ecological constraint via an unavailable residue fraction that is left on-site for ecological functions. The required size of this fraction has been investigated (Daioglou et al. 2015), but is dependent on local circumstances and requires additional understanding. The unavailable fraction currently used in IAMs or residue availability estimations may thus be insufficient to guarantee sustainability and lead to overestimation of sustainable residue supply potential (Searle and Malins 2015).

Several potential effects and implications of large-scale residue use for bioenergy also require further research. First, the life-cycle environmental impacts of collecting, processing, transporting, and using biomass residues for energy may be significant. Biogenic carbon emissions from bioenergy, which are considered GHG neutral in IAMs, should be included here, for instance using time-integrated metrics (Cherubini et al. 2011, 2016). Furthermore, it would be interesting to analyse the environmental consequences of using residues for bioenergy rather than for other potential purposes, including feed, fibre, construction materials and bio-char, or letting them decompose (as studied for forestry residues by Repo et al. 2012, Gustavsson et al. 2015, and Hanssen et al. 2017). Similarly, the economic consequences of taking residues off the field or diverting them from other sectors towards bioenergy require further exploration. Utilising available residues may increase agricultural/forestry profitability (e.g. Smeets et al. 2015) and production. Diverting residues from other sectors may come with significant opportunity costs (Carriquiry et al. 2011). From a more theoretical perspective, increased utilisation and valorisation of residues could justify shifting part of the environmental burden of agricultural and forestry products (i.e. food or timber) towards agricultural and forestry residues, for instance through economic allocation.

## Conclusions

We conclude the following:

- Based on the results of eight IAMs, this study shows that residues might cost-competitively play a large role in the twenty-first century bioenergy supply. At high bioenergy demand, which was exogenously forced in this study, residues could meet 7–50% of bioenergy demand towards 2050 and 2–30% towards 2100. When also considering (mean) literature-estimated residue availability, residues could provide around 55 EJ/year by 2050.
- IAM results vary widely at the global scale and especially at the regional scale. Inter-model variation arises mainly from (1) model structure, where endogenous supply potential and curves better capture residue supply dynamics; (2) modelling of agricultural and forestry production, which can be further harmonised to match scenario storylines; (3) definitions of residues; and (4) residue supply constraints and residue cost components, which can be modelled in more detail.
- Despite inter-model variation, the patterns and drivers of residue supply and underlying cost dynamics are similar across IAMs. Residues supply the majority of bioenergy at low bioenergy demand. With higher demand or biomass prices, the quantity of residues supplied for energy increases. However, as available residues are depleted, the share of residues in total bioenergy still decreases.
- In the studied IAMs, GHG emission pricing and land protection can increase the costs of using land for lignocellulosic bioenergy crop cultivation, leading to a disproportional increase in the costs of (land-intensive) lignocellulosic bioenergy crops and therefore increased residue use and a larger share of bioenergy being covered by residues.
- The important role of residues in IAM projections of bioenergy use largely fits within current estimates of residue availability. However, logistic and sustainability *constraints*, as well as economic and environmental *implications* of large-scale residue use for bioenergy, need to be addressed in future research.

## Electronic supplementary material

ESM 1 (DOCX 1278 kb)

## Footnotes

1 Modern bioenergy includes electricity, heat, or transport fuels derived from biomass; it is distinguished from traditional biomass use for heating and cooking in individual households in developing countries (Smith et al. 2014).

2 The technical/available potential, i.e. accounting for ecological constraints (i.e. preserving soil quality, carbon storage, and biodiversity) and economic constraints (i.e. alternative uses of residues), see section 2.4.

3 These residue supply curves set what quantity of residues can be supplied at what costs. Together with the price of biomass used for energy, they determine what quantity of the available residue potential is supplied for bioenergy.

4 For example, current natural protected areas. The supply overview discusses the default land protection assumptions in the models as well as provide an illustration of their implications for biomass supplies (Rose et al. this issue)

5 Variation is reported here as the maximum percentage above and below inter-model mean in 2100.

6 All models except NLU, which does not contain a residue supply curve and in which all residues available for energy are always used, and DNE21+, in which the amount of residues available for energy, are limited and already depleted at low demand.

7 GLOBIOM and Lauri et al. (2014) include beyond harvesting and processing residues: recycled wood, stump removal, and additional roundwood extraction for bioenergy—partly incentivised by co-benefits of selling residues. This explains their large availability and supplied quantity of forestry residues.

8 For example, more alternative uses of residues that compete with utilisation for bioenergy, or including the effects of residue removal on soil fertility and carbon stocks.
