# Supplementary material for: Biomass residues as twenty-first century bioenergy feedstock—a comparison of eight integrated assessment models
Source: Clim Change. 2019 Sep 10;163(3):1569–86. doi: 10.1007/s10584-019-02539-x (PMC7746566; doi:10.1007/s10584-019-02539-x)
Supplement: Supplementary file 1 — (DOCX 1278 kb) [file 10584_2019_2539_MOESM1_ESM.docx]

**Biomass residues as 21^st^ century bioenergy feedstock**

a comparison of eight integrated assessment models

S.V. Hanssen, V. Daioglou, Z.J.N. Steinmann, S. Frank, A. Popp, T. Brunelle, P. Lauri, T. Hasegawa, M.A.J. Huijbregts, D.P. Van Vuuren

Supporting information

**Table S1** References for the Integrated Assessment Models used in this study

| Integrated Assessment Model | reference(s) with detailed model description |
| --- | --- |
| AIM | Fujimori et al. 2012; Hasegawa et al., 2017 |
| BET | Yamamoto et al., 2014 |
| DNE21+ | Akimoto et al., 2008, 2010 |
| GCAM | Edmonds & Reilly, 1985; Kim et al., 2006 |
| GLOBIOM^a^ | Havlik et al., 2014; Lauri et al., 2014 |
| GRAPE | Kurosawa, 1999; Kurosawa et al., 2006 |
| IMAGE | Stehfest et al., 2014 |
| NLU^a^ | Souty et al. 2012 |

Notes: ^a^ The NLU and GLOBIOM models are not IAMs *sensu stricto*, but rather land use competition models and IAM components that focus on agriculture and forestry, respectively.

**Table S2** Subset of EMF-33 scenarios used in this study

| Scenario name | Exogenous 2^nd^ gen. bioenergy demand | Exogenous  biomass price | Additional scenario  components |
| --- | --- | --- | --- |
| B100 B200 B300 B400 | model baseline demand in 2010 linearly increases to 100, 200, 300 or 400 EJ/yr by 2100 | *n/a* | *n/a* |
| B100C B200C  B300C B400C | as above | *n/a* | *GHG price*: 20 US$_2005_/tonne CO_2_eq. in 2020, with a 3% annual increase^a^ |
| B100LP B200LP B300LP B400LP | as above | *n/a* | *Land protection*: default land protection settings per model^b^ |
| B100CLP B200CLP B300CLP B400CLP | as above | *n/a* | *GHG price* and *Land protection*  (as above) |
| PB3 PB5 PB9 PB15 | *n/a* | price fixed at 3/5/9/15 US$_2005_ /GJ at farm gate | *n/a* |
| PB3C PB5C,  PB9C PB15C | *n/a* | as above | *GHG price*: 20 US$_2005_/tonne CO_2_eq. in 2020, with a 3% annual increase^a^ |

Notes: **^a^** this GHG price is applied to all major GHGs (CO_2_, CH_4_ and N_2_O) and affects all modelled GHG emission mitigation technologies; **^b^** Land protection means that on top of the current natural protected areas, certain areas are to remain or transform in(to) a natural state and are not available for human land uses such as agriculture, model default land protection settings determine what areas (Rose et al., *this issue*).

**Table S3** Aggregation at five region level (IIASA, 2017)

| Region | Countries |
| --- | --- |
| **OECD90** = OECD member countries in 1990. | Australia, Austria, Belgium, Canada, Denmark, Fiji, Finland, France, French Polynesia, Germany, Greece, Guam, Iceland, Ireland, Italy, Japan, Luxembourg, Netherlands, New Caledonia, New Zealand, Norway, Portugal, Samoa, Solomon Islands, Spain, Sweden, Switzerland, Turkey, United Kingdom, United States of America, Vanuatu |
| **REF** = Countries from the Reforming Economies of Eastern Europe and the Former Soviet Union. | Albania, Armenia, Azerbaijan, Belarus, Bosnia and Herzegovina, Bulgaria, Croatia, Cyprus, Czech Republic, Estonia, Georgia, Hungary, Kazakhstan, Kyrgyzstan, Latvia, Lithuania, Malta, Poland, Republic of Moldova, Romania, Russian Federation, Slovakia, Slovenia, Tajikistan, TFYR Macedonia, Turkmenistan, Ukraine, Uzbekistan, Yugoslavia |
| **ASIA** = The region includes most Asian countries with the exception of the Middle East, Japan and Former Soviet Union states. | Afghanistan, Bangladesh, Bhutan, Brunei Darussalam, Cambodia, China, China Hong Kong SAR, China Macao SAR, Democratic People's Republic of Korea, East Timor, India, Indonesia, Lao People's Democratic Republic, Malaysia, Maldives, Mongolia, Myanmar, Nepal, Pakistan, Papua New Guinea, Philippines, Republic of Korea, Singapore, Sri Lanka, Taiwan, Thailand, Viet Nam |
| **MAF** = This region includes the countries of the Middle East and Africa. | Algeria, Angola, Bahrain, Benin, Botswana, Burkina Faso, Burundi, Cameroon, Cape Verde, Central African Republic, Chad, Comoros, Congo, Cote d'Ivoire, Democratic Republic of the Congo, Djibouti, Egypt, Equatorial Guinea, Eritrea, Ethiopia, Gabon, Gambia, Ghana, Guinea, Guinea-Bissau, Iran (Islamic Republic of), Iraq, Israel, Jordan, Kenya, Kuwait, Lebanon, Lesotho, Liberia, Libyan Arab Jamahiriya, Madagascar, Malawi, Mali, Mauritania, Mauritius, Morocco, Mozambique, Namibia, Niger, Nigeria, Oman, Qatar, Reunion, Rwanda, Saudi Arabia, Senegal, Sierra Leone, Somalia, South Africa, Sudan, Swaziland, Syrian Arab Republic, Togo, Tunisia, Uganda, United Arab Emirates, United Republic of Tanzania, Western Sahara, Yemen, Zambia, Zimbabwe |
| **LAM** = This region includes the countries of Latin America and the Caribbean. | Argentina, Bahamas, Barbados, Belize, Bolivia, Brazil, Chile, Colombia, Costa Rica, Cuba, Dominican Republic, Ecuador, El Salvador, Guadeloupe, Guatemala, Guyana, Haiti, Honduras, Jamaica, Martinique, Mexico, Netherlands Antilles, Nicaragua, Panama, Paraguay, Peru, Puerto Rico, Suriname, Trinidad and Tobago, Uruguay, Venezuela |

**Table S4** ANOVA-based variance decomposition analysis results indicating how exogenous bioenergy demand, GHG pricing and inter-model differences contribute to differences in the quantity of residues supplied across all studied models and exogenous demand/GHG pricing scenarios, the interaction effect of bioenergy demand and GHG pricing was negligible (<0.7% attributed variance)

| Dependent variable  *(models included)* | Factor | Variance explained | |
| --- | --- | --- | --- |
|  | *Year:* | 2050 | 2100 |
| Quantity of residues supplied - log transformed *(all models)* | exogenous bioenergy demand | 8.6% | 5.0% |
|  | presence of GHG pricing | 0.5% | 2.3% |
|  | inter-model differences (residual) | 90.9% | 92.8% |
| Residues as share of bioenergy supply -  logit transformed *(all models)* | exogenous bioenergy demand | 10.9% | 14.8% |
|  | presence of GHG pricing | 0.1% | 3.2% |
|  | inter-model differences (residual) | 89.0% | 81.8% |
| Quantity of residues supplied - log transformed *(excl. DNE21+ and NLU)* | exogenous bioenergy demand | 15.6% | 25.1% |
|  | presence of GHG pricing | 0.9% | 11.5% |
|  | inter-model differences (residual) | 83.4% | 63.4% |
| Residues as share of bioenergy supply  - logit transformed *(excl. DNE21+ and NLU)* | exogenous bioenergy demand | 11.7% | 23.9% |
|  | presence of GHG pricing | 0.1% | 10.6% |
|  | IAM differences (residual) | 88.2% | 64.8% |

**
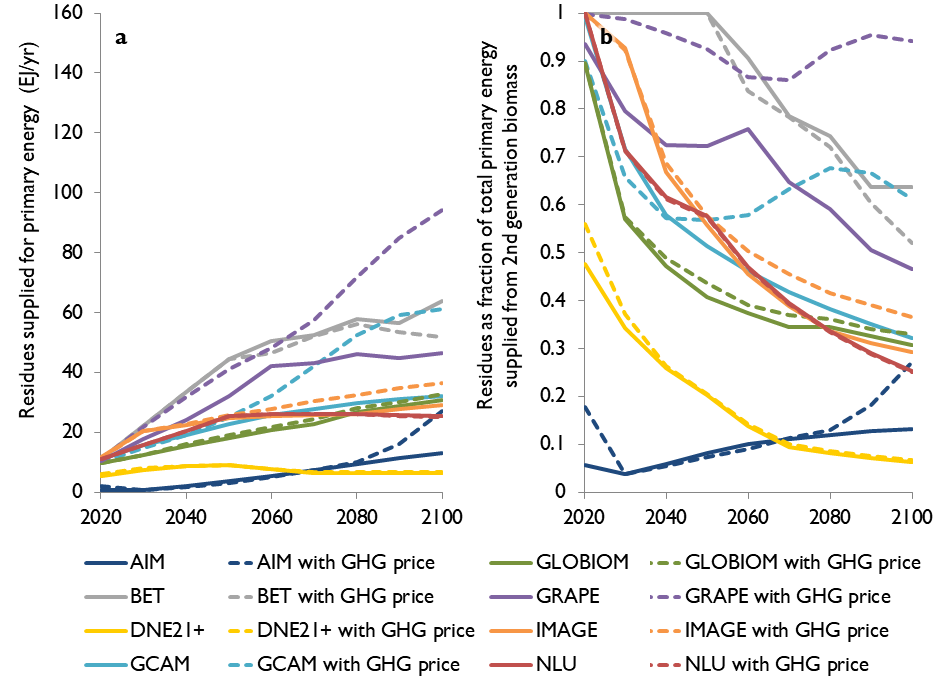
**

**Figure S1** Quantity of residue supplied for primary energy (EJ/year) at an exogenous demand for 2^nd^ generation bioenergy that increases linearly from 2010 levels to 100 EJ/yr by 2100, with and without GHG pricing (scenarios B100 and B100C respectively; see Table S2) (**a**), residues as share of total second-generation biomass use for primary energy under the same scenarios (**b**), for NLU and DNE21+ dashed lines may underlie their respective solid line


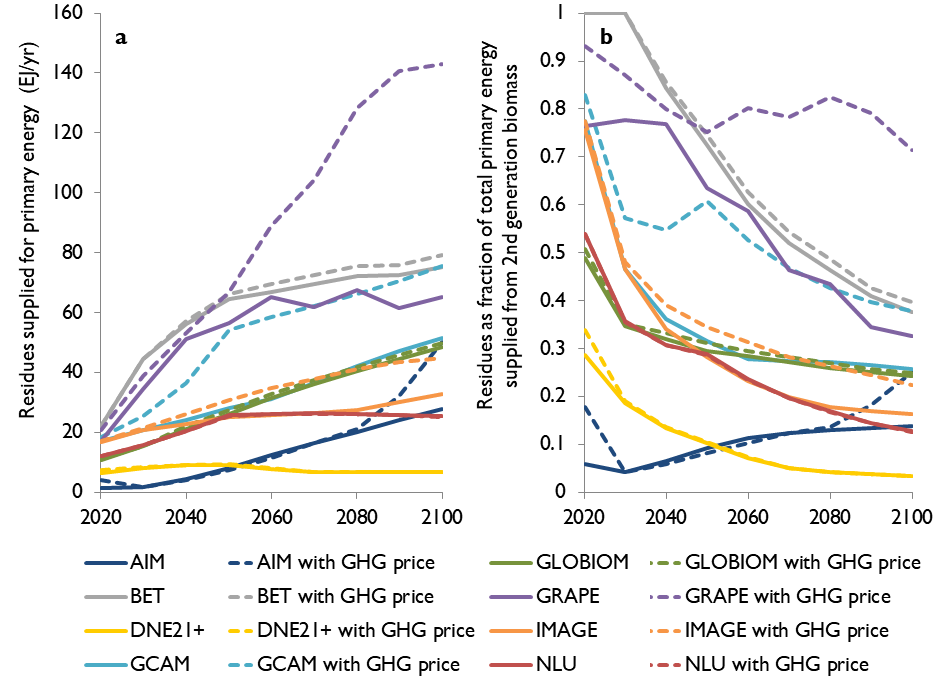


**Figure S2** Quantity of residue supplied for primary energy (EJ/year) at an exogenous demand for 2^nd^ generation bioenergy that increases linearly from 2010 levels to 200 EJ/yr by 2100, with and without GHG pricing (scenarios B200 and B200C respectively; see Table S2) (**a**), residues as share of total second-generation biomass use for primary energy under the same scenarios (**b**), for NLU and DNE21+ dashed lines may underlie their respective solid line

**
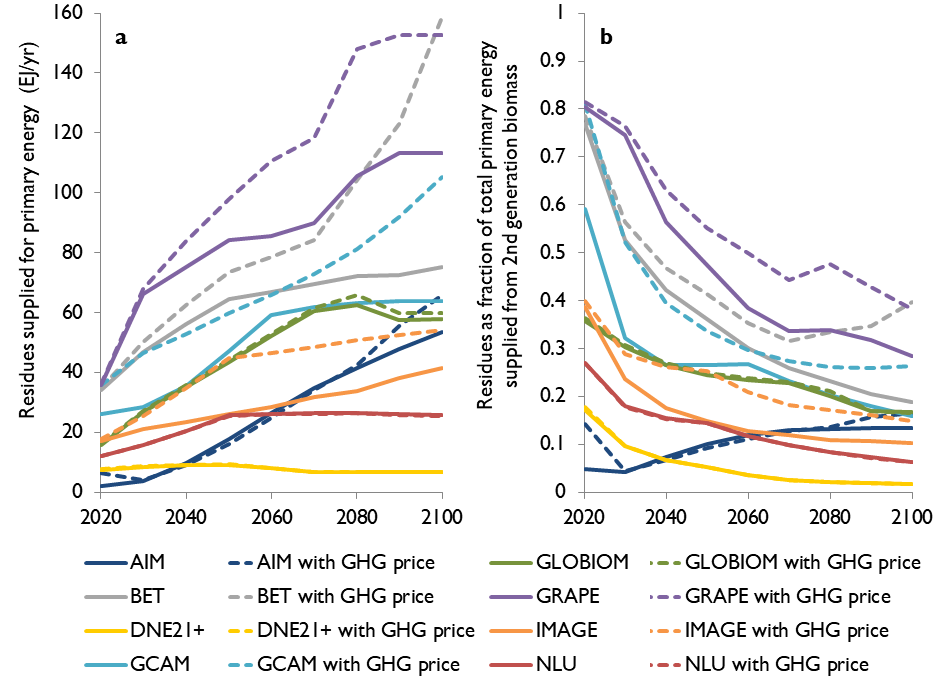
**

**Figure S3** Quantity of residue supplied for primary energy (EJ/year) at an exogenous demand for 2^nd^ generation bioenergy that increases linearly from 2010 levels to 400 EJ/yr by 2100, with and without GHG pricing (scenarios B400 and B400C respectively; see Table S2) (**a**), residues as share of total second-generation biomass use for primary energy under the same scenarios (**b**), for NLU and DNE21+ dashed lines may underlie their respective solid line

**
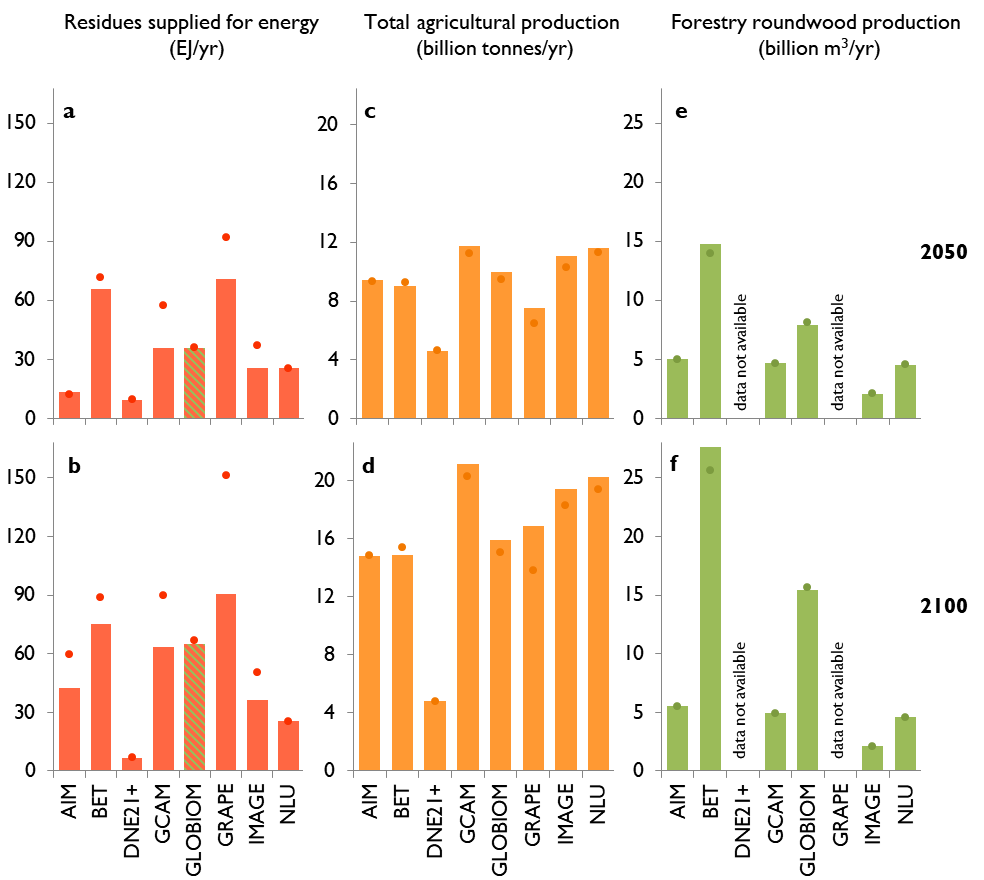
**

**Figure S4** Quantity of residue supplied for primary energy (EJ/year) in 2050 (**a**) and 2100 (**b**), agricultural production (billion tonnes) in 2050 (**c**) and 2100 (**d**), and roundwood production (billion m^3^) in 2050 (**e**) and 2100 (**f**) in a scenario with an exogenous demand for 2^nd^ generation bioenergy that increases linearly from 2010 levels to 300 EJ/yr by 2100, dots indicate the level of supplied energy, agricultural production and roundwood production when GHG pricing is included in the scenario, note that GLOBIOM only includes forestry residues

**
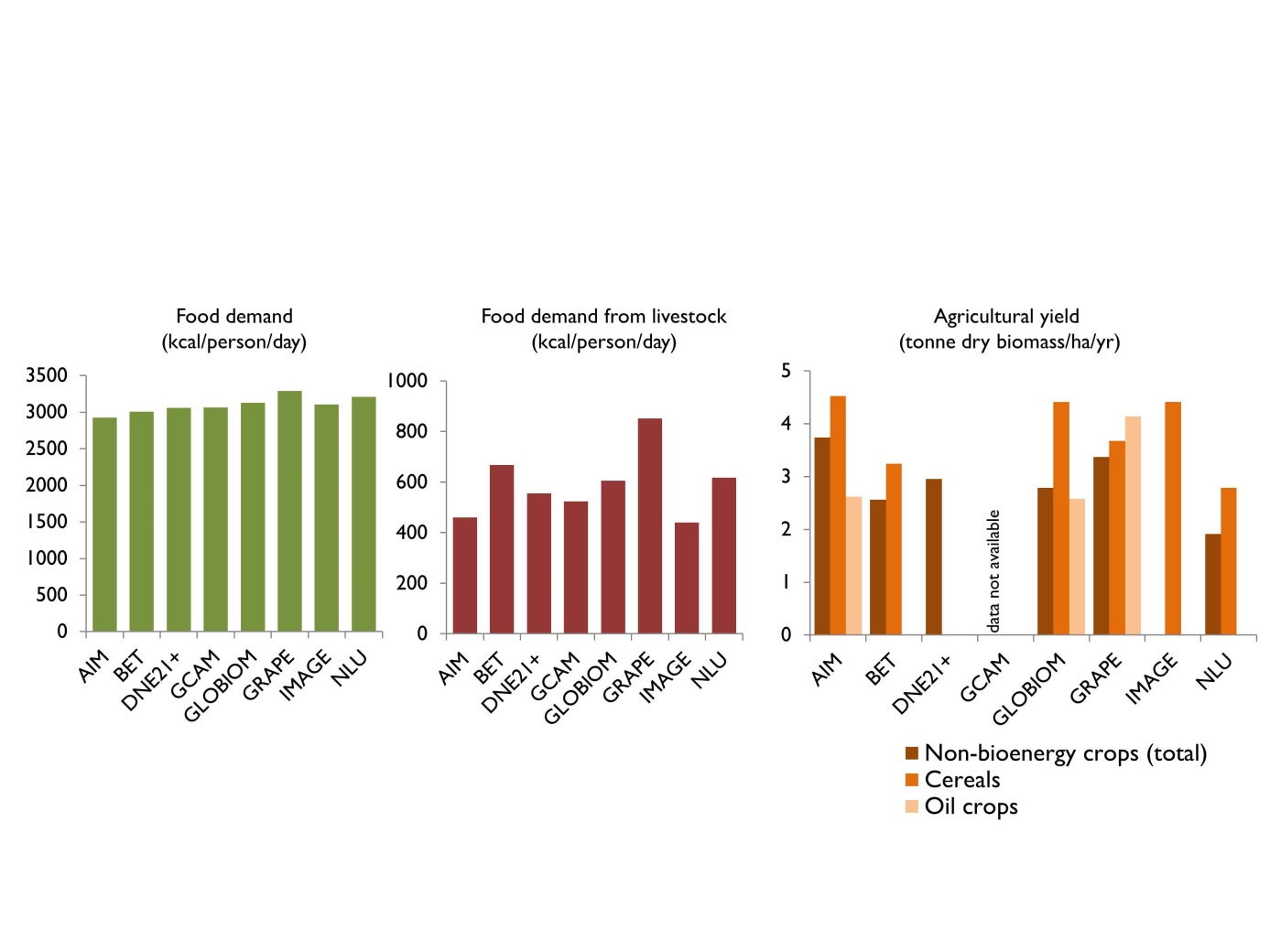
**

**Figure S5** Model outcomes for 2050 in a scenario with an exogenous demand for 2^nd^ generation bioenergy that increases linearly from 2010 levels to 300 EJ/yr by 2100: per capita food demand (**a**), diet, i.e., per capita food demand from livestock (meat, dairy) (**b**), and agricultural yields for total non-bioenergy crops, as well as for cereals and oil crops, separately (**c**).

*
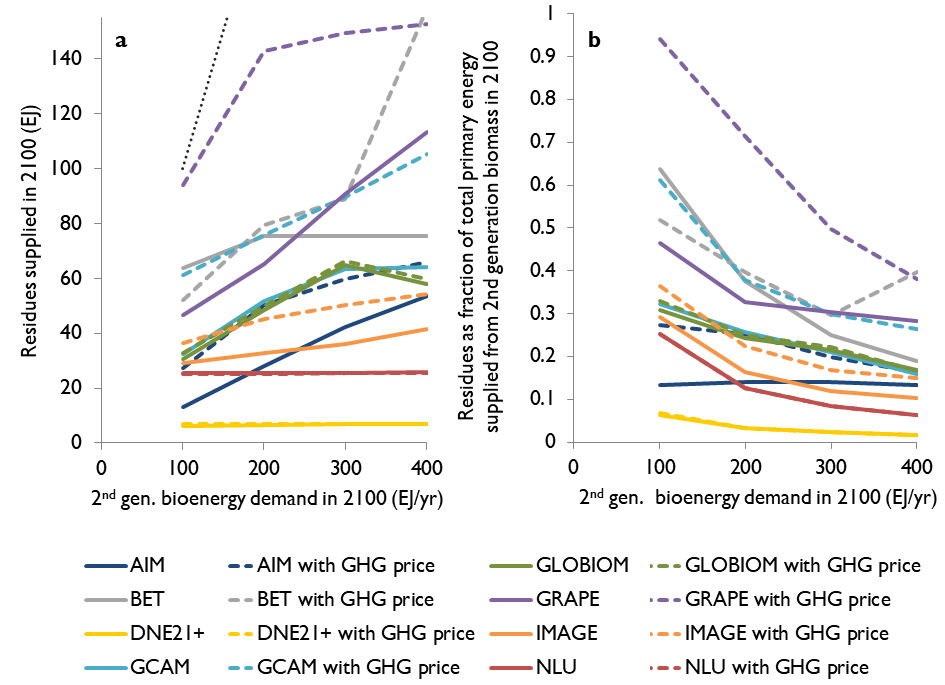
*

**Figure S6** Quantity of residues supplied in the studied IAMs for the year 2100, across four scenarios with increasing exogenous bioenergy demand (to 100, 200, 300 and 400 EJ/yr by 2100; see Table S2), with and without GHG pricing (**a)**, residues as share of total second-generation biomass use for primary energy across the same scenarios in 2100 (**b**), the black dotted line indicates residues meeting 100% of exogenous bioenergy demand


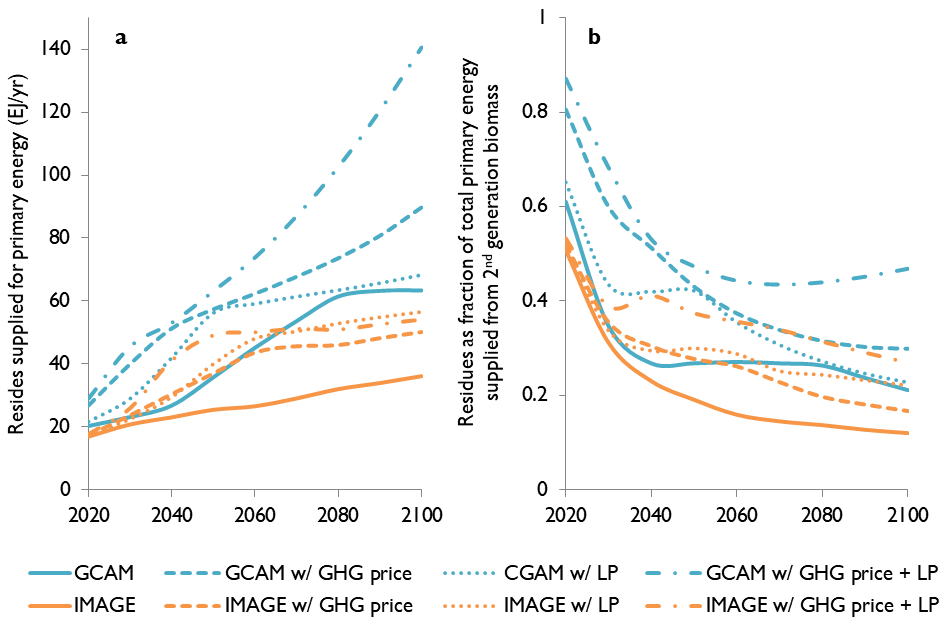


**Figure S7** Quantity of Residues supplied for primary energy (EJ/year), with and without land protection (LP), and with and without GHG emissions pricing, at an exogenous demand for 2^nd^ generation bioenergy that increases linearly from 2010 levels to 300 EJ/yr by 2100 (**a**), residues as share of total second-generation biomass use for primary energy under the same scenarios (**b**)

*
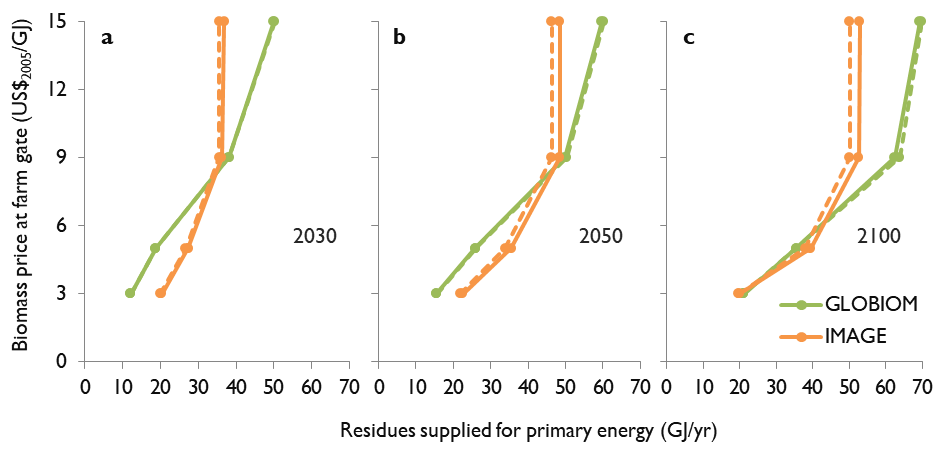
*

**Figure S8** Quantity of residues supplied for primary energy in 2030 (**a**), 2050 (**b**), and 2100 (**c**), at fixed exogenous biomass prices of 3, 5, 9 and 15 US$_2005_/GJ second-generation biomass (specifically used for energy) at farm gate, with (dashed) and without (no dash) GHG pricing

*
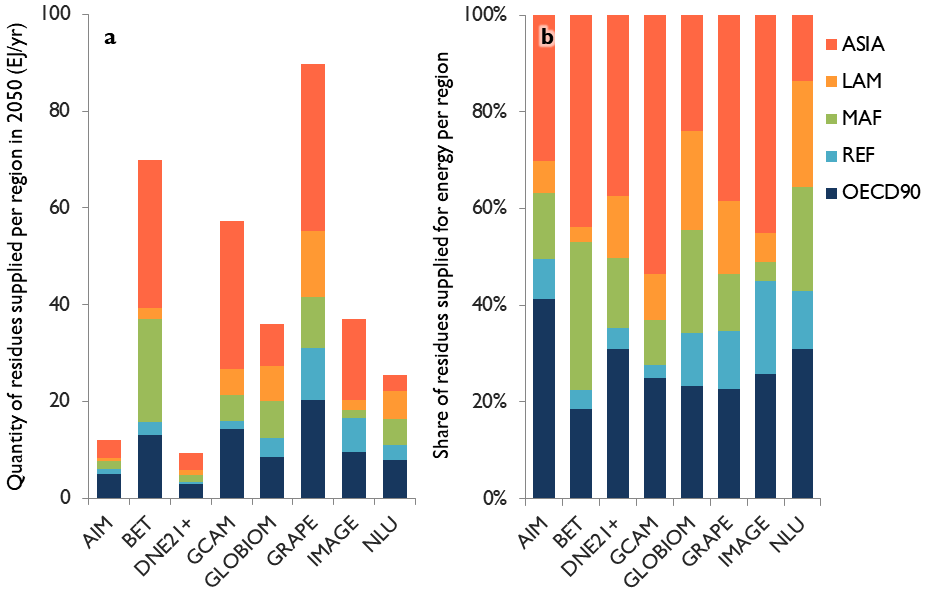
*

**Figure S9** Quantity of biomass residues supplied for energy per region in 2050 in a scenario with an exogenous bioenergy demand of 300 EJ/yr by 2100 and GHG pricing (**a**), share of residues supplied for energy per region in 2050 (**b**), abbreviations: LAM= Latin America, MAF= Middle East and Africa; REF = reforming economies (former Soviet Union and Eastern Europe); OECD90 = OECD member countries in 1990, for regional definitions see Table S3


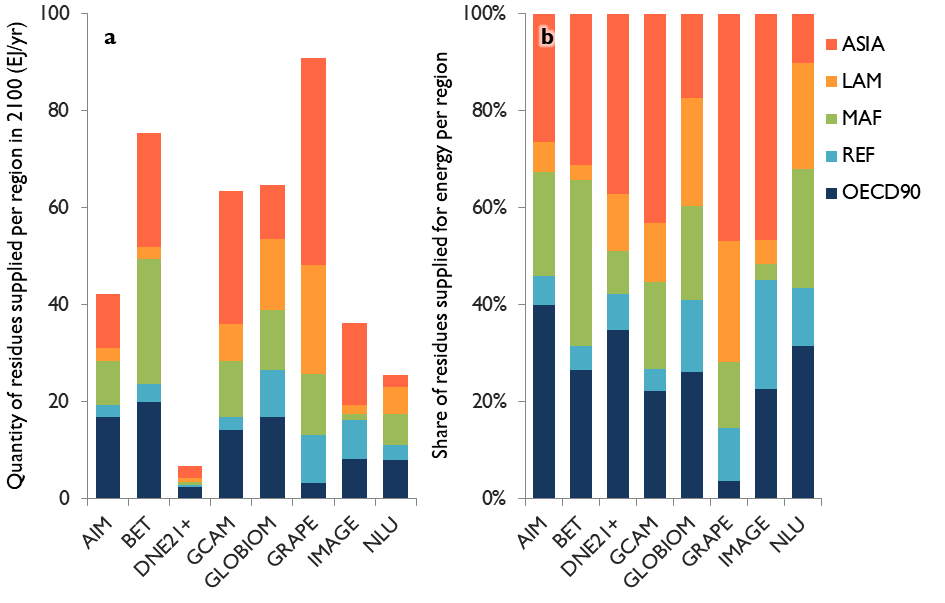


**Figure S10** Quantity of biomass residues supplied for energy per region in 2100 in a scenario with an exogenous bioenergy demand of 300 EJ/yr by 2100 (**a**), share of residues supplied for energy per region in 2100 (**b**), abbreviations: LAM= Latin America, MAF= Middle East and Africa; REF = reforming economies (former Soviet Union and Eastern Europe); OECD90 = OECD member countries in 1990, for regional definitions see Table S3


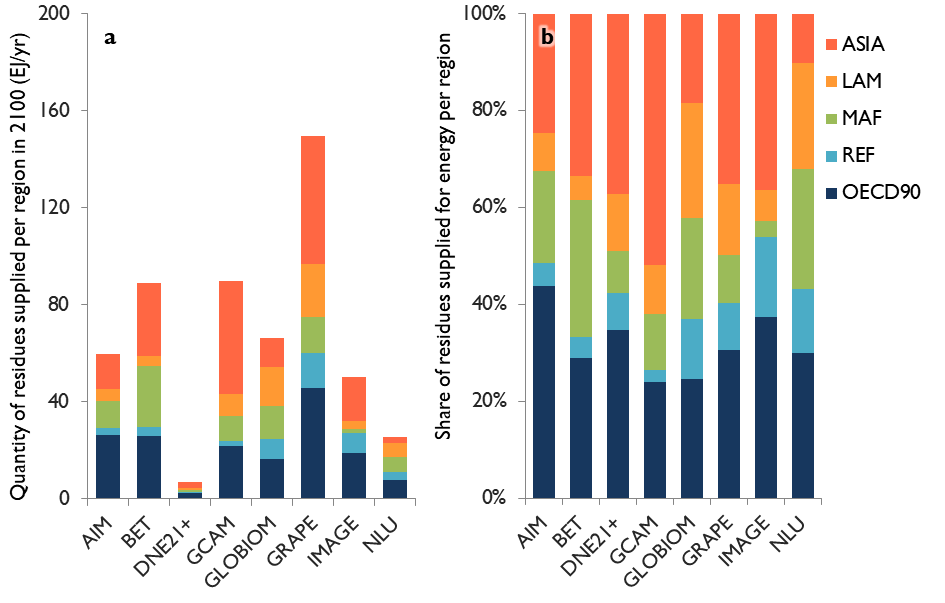


**Figure S11** Quantity of biomass residues supplied for energy per region in 2100 in a scenario with an exogenous bioenergy demand of 300 EJ/yr by 2100 and GHG pricing (**a**), share of residues supplied for energy per region in 2100 (**b**), abbreviations: LAM= Latin America, MAF= Middle East and Africa; REF = reforming economies (former Soviet Union and Eastern Europe); OECD90 = OECD member countries in 1990, for regional definitions see Table S3

**
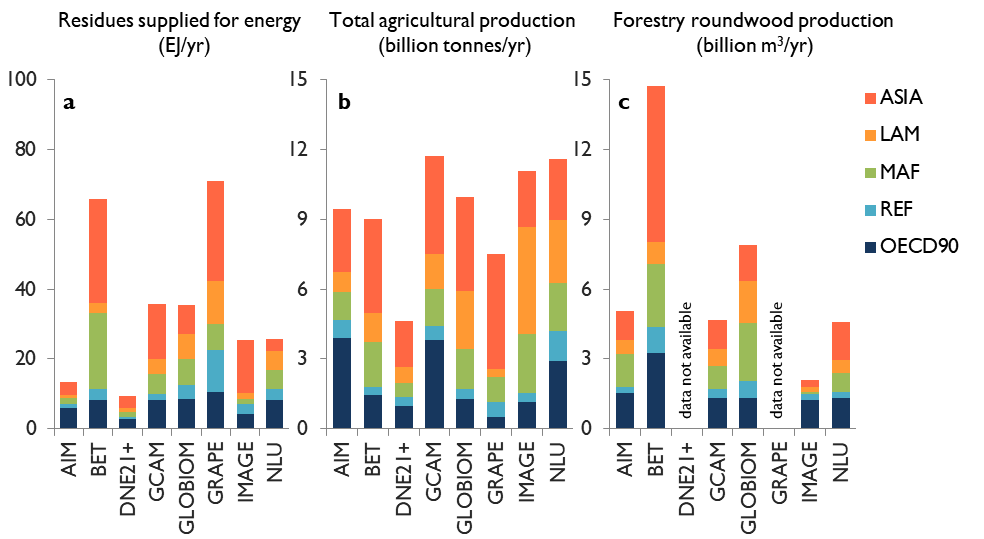
Figure S12**Quantity of residue supplied for primary energy per region (EJ/year) (**a**), agricultural production per region (billion tonnes) (**b**), and roundwood production per region (billion m^3^) (**c**) in a scenario with an exogenous demand for 2^nd^ generation bioenergy that increases linearly from 2010 levels to 300 EJ/yr by 2100, note that GLOBIOM only includes forestry residues, abbreviations: LAM= Latin America, MAF= Middle East and Africa; REF = reforming economies (former Soviet Union and Eastern Europe); OECD90 = OECD member countries in 1990, for regional definitions see Table S3

Supplemental references

Akimoto K, Sano F, Oda J, Homma T, Rout UK, Tomoda T (2008) Global emission reductions through a sectoral intensity target scheme. Climate Policy 8:46–59. doi:10.3763/cpol.2007.0492

Akimoto K, Sano F, Homma T, Oda J, Nagashima M, Kii M (2010) Estimates of GHG emission reduction potential by country, sector, and cost. Energy Policy 38:3384–3393. doi:10.1016/j.enpol.2010.02.012

Edmonds J, Reilly J (1985) Global Energy – Assessing the Future. Oxford University Press, Oxford

Fujimori S, Masui T, Matsuoka Y (2012) AIM/CGE Basic Manual. Discssion Paper Series, Center for Social and Environmental Systems Research, NIES, Tsukuba, Japan

Hasegawa T, Fujimori S, Ito A, Takahashi K, Masui T (2017) Global land-use allocation model linked to an integrated assessment model. Sci. Total Environ. 580:787-796 doi: https://doi.org/10.1016/j.scitotenv.2016.12.025

Havlik P, Valin H, Herrero M, Obersteiner M, Schmid E, et al. (2014) Climate change mitigation through livestock system transition. Proc. Natl. Acad. Sci. U.S.A. 111:3709-3714 doi: 1308044111

IIASA (2017) EMF33 Bioenergy Scenario Database. International Institute for Applied Systems Analysis https://tntcat.iiasa.ac.at/EMF30BIODB/dsd?Action=htmlpage&page=about Accessed 30 October 2017

Kim SH, Edmonds JA, Lurz J, Smith SJ, Wise MA (2006) The object-oriented energy climate technology systems (ObjECTS) framework and hybrid modeling of transportation in the MiniCAM long-term, global integrated assessment model. Energy Journal 27:63-92.

Kurosawa A (2006). Multigas mitigation: an economic analysis using GRAPE model. Energy Journal Special Issue on Multi-Greenhouse Gas Mitigation and Climate Policy 27:275-288.

Kurosawa A, Yagita H, Zhou WS, Tokimatsu K, Yanagisawa Y (1999) Analysis of carbon emission stabilization targets and adaptation by integrated assessment model. Energy Journal Kyoto Special Issue 20:157-175.

Lauri P, Havlik P, Kindermann G, Forsell N, Böttcher H, Obersteiner M (2014) Woody biomass energy potential in 2050. Energy Policy 66:19–31. doi: 10.1016/j.enpol.2013.11.033

Rose S, et al (this issue) Global biomass supply modelling for long-run management of the climate system. Clim Change EMF-33 Special Issue

Souty F, Brunelle F, Dumas P, Dorin B, Ciais P, Crassous R, Müller C, Bondeau A (2012) The nexus land-use model version 1.0, an approach articulating biophysical potentials and economic dynamics to model competition for land-use. Geosci Model Dev 5:1297-1322. doi:10.5194/gmd-5-1297-2012

Stehfest E, van Vuuren D, Kram T, Bouwman T, Alkemade R, Bakkenes M et al. (2014) Integrated Assessment of Global Environmental Change with IMAGE 3.0. Model Description and Policy Applications. PBL Netherlands Environmental Assessment Agency, The Hague.

Yamamoto H, Sugiyama M, Tsutsui J (2014) Role of end-use technologies in long-term GHG reduction scenarios developed with the BET model. Clim Change 123:583-596 doi: 10.1007/s10584-013-0938-6
